# Heterogeneity of Ovarian Theca and Interstitial Gland Cells in Mice

**DOI:** 10.1371/journal.pone.0128352

**Published:** 2015-06-03

**Authors:** Kanako Miyabayashi, Kaori Tokunaga, Hiroyuki Otake, Takashi Baba, Yuichi Shima, Ken-ichirou Morohashi

**Affiliations:** Department of Molecular Biology, Graduate School of Medical Sciences, Kyushu University, Fukuoka, Fukuoka, Japan; East Carolina University, UNITED STATES

## Abstract

It has been established that two developmentally and functionally distinct cell types emerge within the mammalian testis and adrenal gland throughout life. Fetal and adult types of steroidogenic cells (i.e., testicular Leydig cells and adrenocortical cells) develop in the prenatal and postnatal period, respectively. Although the ovary synthesizes steroids postnatally, the presence of fetal-type steroidogenic cells has not been described. We had previously established transgenic mouse lines in which fetal Leydig cells were labeled with an EGFP reporter gene by the FLE (fetal Leydig enhancer) of the *Ad4BP/SF-1* (*Nr5a1*) gene. In the present study, we examined the reporter gene expression in females and found that the reporter gene is turned on in postnatal ovaries. A comparison of the expressions of the EGFP and marker genes revealed that EGFP is expressed in not all but rather a proportion of steroidogenic theca and in interstitial gland cells in the ovary. This finding was further supported by experiments using BAC transgenic mice in which reporter gene expression recapitulated endogenous *Ad4BP/SF-1* gene expression. In conclusion, our observations from this study strongly suggest that ovarian theca and interstitial gland cells in mice consist of at least two cell types.

## Introduction

Similar to the testis, the ovary exerts two predominant functions. One is the production of mature germ cells, while the other is the synthesis of sex steroids, which are thereafter delivered to the whole body and thereby induce female-specific characteristics. Estradiol, a potent estrogen, is synthesized in the ovary by successive reactions mediated by five enzymes: cholesterol side-chain cleavage cytochrome P450scc (CYP11A), 3β-hydroxysteroid dehydrogenase (3β-HSD), 17alpha-hydroxylase/17, 20-lyase cytochrome P450 (CYP17), 17β-hydroxysteroid dehydrogenase (17β-HSD), and aromatase P450 (CYP19) [1, 2]. Interestingly, the former three enzymes are expressed within theca cells surrounding follicles while the latter two enzymes are within the granulosa cells inside follicles, indicating that estradiol—the final product of the sex steroid synthetic pathway in the ovary—is produced by cooperative actions between the two distinct cell types. In addition to theca and granulosa cells, the interstitial gland cells in ovaries of rodent species are steroidogenic as well, possessing functions similar to those of theca cells [3].

Ad4BP/SF-1 (NR5A1), a member of a nuclear receptor superfamily, was originally identified as a factor that regulates steroidogenic *CYP* gene expression in the adrenal cortex [4–6]. Evidence accumulated in subsequent studies has demonstrated that Ad4BP/SF-1 targets all steroidogenic genes required for the syntheses of gonadal steroids as well as of corticosteroids [7, 8]. This functional relevance to steroidogenesis in the gonads is supported by the distribution of Ad4BP/SF-1, which is enriched in steroidogenic cells, such as Leydig cells in the testis and theca and interstitial gland cells in the ovary. In addition to its involvement to steroidogenesis, the functions of Ad4BP/SF-1 have been discussed from a developmental aspect, since mice in which its gene expression is disrupted develop neither adrenal glands nor gonads [9–11]. As a possible reason for this tissue agenesis, a recent study revealed that *Ad4BP/SF-1* is involved in a variety of biological processes, [12] such as energy metabolism, through regulating glycolytic genes [13]. These findings raised a possibility that disruption of the *Ad4BP/SF-1* gene leads to aberrant cellular functions and thereby results in this striking phenotype.

Developmentally, the male and female gonads in mammals start to functionally differentiate in the fetal and postnatal ages, respectively. Sertoli and Leydig cells emerge in the fetal testis, whereas ovarian theca and granulosa cells differentiate after birth in mice. The differences between the Leydig cells that emerge in the fetal testis and the Leydig cells in the adult testis have been discussed in terms of their morphological and functional features [14]. Studies that successfully distinguished these cell types based on differential gene expressions support this notion [15–18]. Moreover, a recent study clearly demonstrated that fetal Leydig cells (FLCs) and adult Leydig cells (ALCs) can be distinguished by how their enhancers of the *Ad4BP/SF-1* gene are utilized [19]. A DNA fragment that can induce gene expression in FLCs but not ALCs was isolated from the gene. In subsequent transgenic mouse experiments involving a construct carrying an EGFP (enhanced green fluorescence protein) reporter gene under the control of the fetal Leydig enhancer (FLE), EGFP was expressed only in FLCs, but not in ALCs.

In the present study, we discovered that the FLE is activated in a population of steroidogenic theca and interstitial gland cells in postnatal mouse ovaries. These EGFP-positive cells were all positive for Ad4BP/SF-1, whereas only a subpopulation of Ad4BP/SF-1-positive cells was positive for EGFP. These observations provide evidence for the first time that the ovary, similar to the testis, contains two cell types in theca and interstitial gland cells.

## Materials and Methods

### DNA construction and generation of transgenic mice

**Fig 1:**
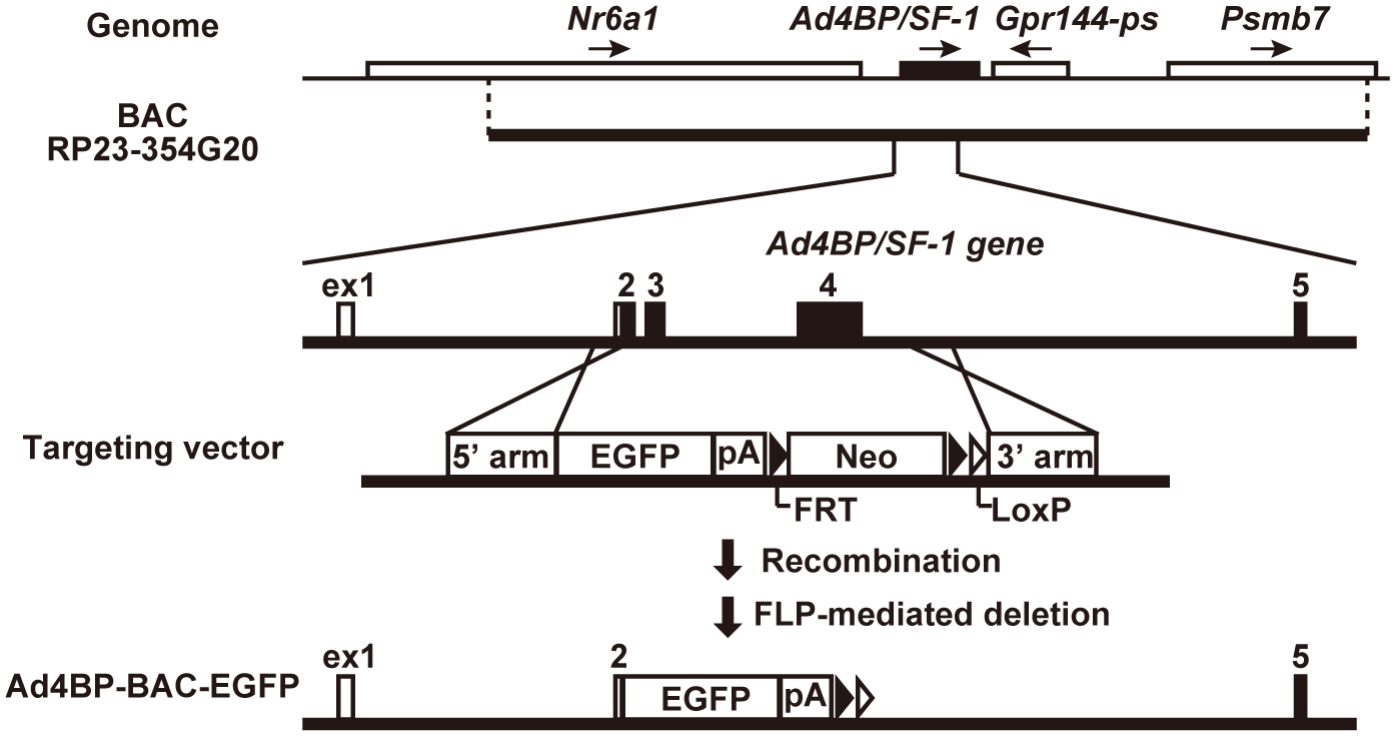
Construction of BAC transgene. Construction of the BAC transgene is shown. The BAC clone (RP23-354G20) used in this study contained *Nr6a1*, *Gpr144-ps*, and *Psmb7* genes in addition to *Ad4BP/SF-1* as indicated at the top. The directions of gene transcription are indicated by arrows. *Ad4BP/SF-1* in the BAC was replaced using the targeting vector by recombination using the 5’ and 3’ arms. Ad4BP-BAC-EGFP was finally obtained by FLP-mediated deletion of the 3’ segment of the targeting vector integrated into the BAC. Detailed procedures are described in the Materials and Methods. *Nr6a1*, nuclear receptor subfamily 6, group A, member 1 (*Gcnf*); *Gpr144-ps*, G protein-coupled receptor 144, pseudogene; *Psmb7*, proteasome subunit, beta type 7; Neo, neomycin resistant gene; pA, poly A; FRT, flippase recombination target; FLP, flippase; EGFP, enhanced green fluorescence protein.

mFLE-EGFP (mutant FLE-EGFP; referred to as SmAc-1.8-Ad4BP(LBmut)-EGFP in our previous study) transgenic mice have been described previously [20]. mFLE-mCherry was constructed by replacing EGFP with mCherry. The resulting construct was injected into the pronuclei of fertilized eggs to generate transgenic mice as described previously [21, 22]. A bacterial artificial chromosome (BAC) containing approximately 106-kb and 100-kb flanking regions at the 5’ and 3’ ends of the *Ad4BP/SF-1* gene, respectively, was purchased from BACPAC Resources, Children’s Hospital Oakland Research Institute (clone ID RP23-354G20; Oakland, CA, USA), and subjected to Red/ET system-based recombineering (Gene Bridges Gmbh, Heidelberg, Germany) [23–25]. A modification vector for the BAC recombineering was constructed as follows (Fig 1). A 479-bp fragment upstream from the first ATG in the 2nd exon of the *Ad4BP/SF-1* gene was used as the 5’ homologous arm. The 5’ arm-EGFP-polyA fragment was amplified from mFLE-EGFP by PCR using the primer set 5’-GGGGTACCCGAATCTCTCCCAATGTCGT-3’ and 5’-GGAATTCATATTAACGCTTACAATTTACGCGT-3’, which carry *Kpn*I and *Eco*RI sites at their respective 5’ ends. A 499-bp fragment in intron 4 was used as the 3’ homologous arm. This fragment was amplified from the BAC DNA by PCR with the primer set 5’-CGGGATCCAGCAACAGGAAGAACTTTCGAG-3’ and 5’-ATAAGAATGCGGCCGCTAGAAAGGGCCAGTGAGAAAAG-3’, which carry *Bam*HI and *Not*I sites at their respective 5’ ends. The 5’ arm-EGFP-polyA fragment and 3’ arm fragment were digested and inserted into *Kpn*I/*Eco*RI sites and *Bam*HI/*Not*I sites, respectively, of a PL451 FRT (flippase recombination target)-loxP-neo BAC modification vector (gifted from Dr. Neal G. Copeland, Houston Methodist Hospital, TX, USA). The constructed vector was sequenced, and an *EGFP-Neo* cassette was introduced into RP23-354G20 by Red/ET system-based homologous recombination. Finally, the *Neo* cassette was deleted by arabinose-induced flippase (flp)-mediated recombination at FRT sites. The BAC construct (Ad4BP-BAC-EGFP) was purified and injected into fertilized eggs, as previously described [26]. The presence or absence of the transgenes in the animals was determined by PCR, using the primers for *EGFP* [20] or *mCherry* (5’-CGCCGACATCCCCGACTACTT-3’ and 5’- CAGCCCATGGTCTTCTTCTGC-3’). All protocols followed for animal experiments were approved by the Animal Care and Use Committee of Kyushu University (Permit Number: A26-001). Mice were sacrificed after sevoflurane anesthesia, and all efforts were made to minimize suffering.

### Characterization of transgenic mice and immunofluorescence

Tissue samples from the transgenic mice were obtained from the fetal to adult stages. Tissue fluorescence was observed using a SZX16/SZX2-ILLT fluorescent stereomicroscope, (Olympus, Tokyo, Japan). Immunofluorescence was performed as described previously [27]. Cryosections (10 μm) were prepared from the tissues fixed in 4% paraformaldehyde at 4°C overnight. Anti-EGFP rabbit polyclonal antibody (1:1000 dilution; #598; Medical and Biological Laboratories, Nagoya, Japan), anti-EGFP rat monoclonal antibody (1:1000 dilution; #04404–84; Nacalai Tesque, Kyoto, Japan), anti-EGFP chicken polyclonal antibody (1:1000 dilution; #ab13970; abcam, Cambridge, UK), anti-DsRed rabbit polyclonal antibody (for mCherry) (1:200; #632496; Clontech, CA, USA), anti-Ad4BP/SF-1 rabbit polyclonal antibody (1:2000 dilution) [28], anti-Ad4BP/SF-1 rat monoclonal antibody (1:100 dilution) [29], and anti-3β-HSD rabbit polyclonal antibody (1:2000 dilution) [26] were used as primary antibodies. Alexa Fluor488 goat anti-rabbit IgG, Alexa Fluor488 goat anti-rat IgG, Alexa Fluor555 goat anti-rabbit IgG, Alexa Fluor555 goat anti-rat IgG (1:500 dilution; Life Technologies, Carlsbad, CA, USA), and Alexa Fluor488 goat anti-chicken IgY (1:500 dilution; #ab150169; abcam, Cambridge, UK) were used as secondary antibodies. 4’6’-diamidino-2-phenylindole (DAPI) (1 μg/ml; SIGMA, St. Louis, MO, USA) was used for nuclear staining. A BZ-9000 microscope (Keyence, Osaka, Japan) was used for the fluorescence imaging experiments.

## Results

### EGFP expression driven by the FLE of *Ad4BP/SF-1* gene in the ovary

In our previous study, we established transgenic mouse lines in which an *EGFP* reporter gene is expressed under the control of the FLE together with the proximal upstream region of *Ad4BP/SF-1* (SmAc-1.8-Ad4BP(LBmut)-EGFP [20]. In this reporter gene construct, an *Lhx*9 binding site localized at the proximal upstream region was mutated (mFLE) in order to abolish the weak EGFP expression in interstitial cells other than FLCs of the fetal testis, as well as that in somatic cells of sexually indifferent gonads of both sexes. This construct will henceforth be referred to as mFLE-EGFP. Since the EGFP-positive cells in the fetal testis were mostly positive for 3β-HSD, this mutation seemed not to affect the expression of EGFP in FLCs.

**Fig 2:**
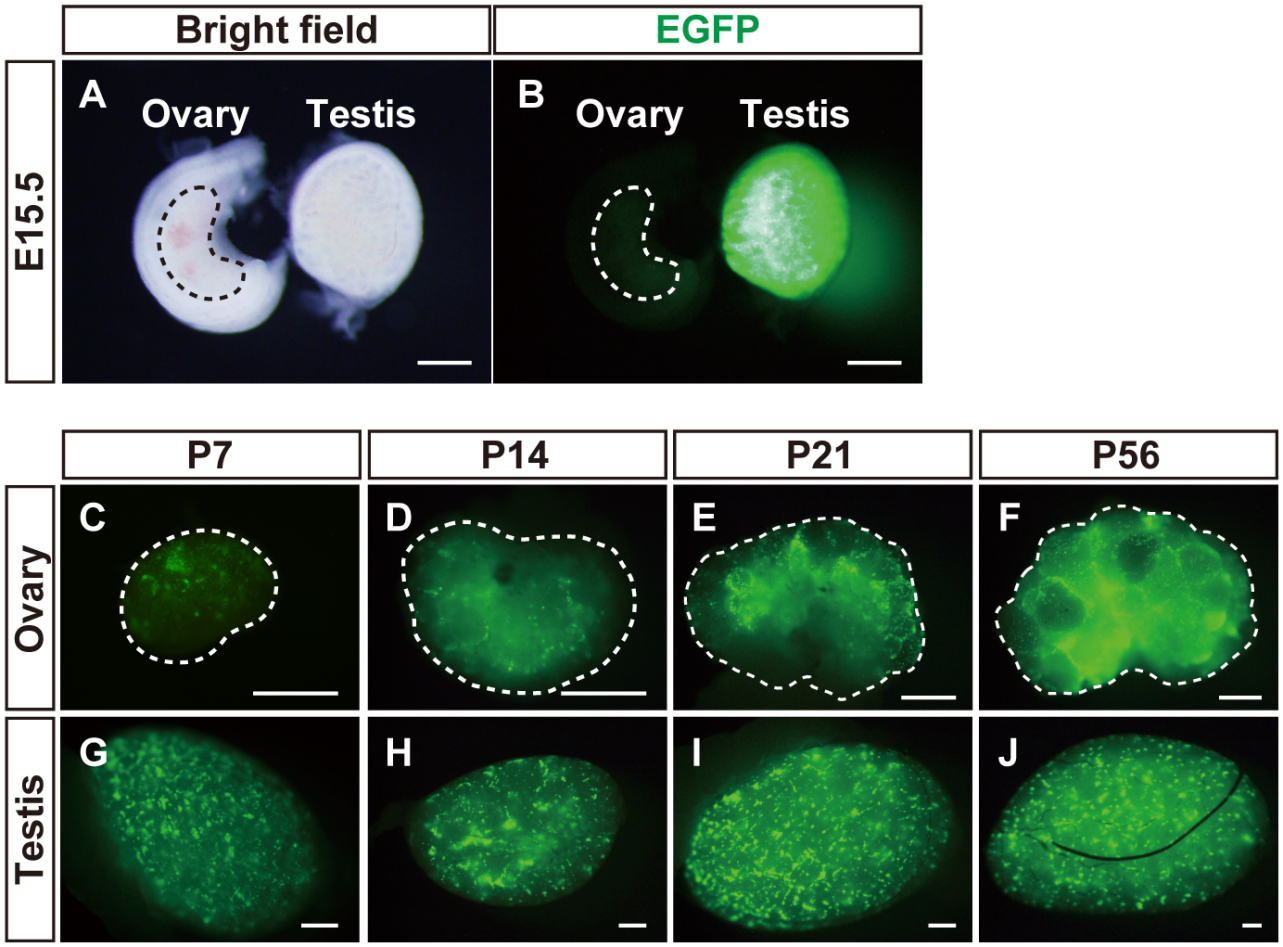
Expression of EGFP induced by FLE in fetal and postnatal gonads. The gonads (ovary and testis) of mFLE-EGFP transgenic mice were prepared in E15.5 (A and B, n = 2), P7 (C and G, n = 4), P14 (D and H, n = 2), P21 (E and I, n = 4), and adult stage at P42 or P56 (F and J, n = 4). Whole views of the gonads are shown. B is a fluorescence view of A. The ovaries are delineated by broken lines. Scale bars = 500 μm.

Although steroidogenic FLCs are differentiated in fetal testes, cells such as fetal theca cells have not been thought to differentiate in the fetal ovaries. Therefore, EGFP reporter gene expression was expected to be undetected in the ovary. In fact, fluorescent microscopic observation showed a clear difference of the EGFP signals between the fetal testis and ovary at E15.5 (Fig 2A and 2B). Careful observation detected a small number of cells displaying very weak EGFP signals in the fetal ovary, although it is unknown whether the expression was induced by the FLE, the basal promoter, or both. Thereafter, clear expression became detectable in postnatal ovaries; EGFP signals could be seen clearly in the ovary at early postnatal day (Fig 2C) and the signal became stronger gradually at P14, at P21 and the adult stage (Fig 2D–2F). With respect to the EGFP expression in the testis, the signal could be detected even in the postnatal period, as previously described [19], indicating that FLCs persist in the postnatal testes (Fig 2G–2J).

### Expression of EGFP in a subpopulation of Ad4BP/SF-1 immunoreactive cells in the ovary

**Fig 3:**
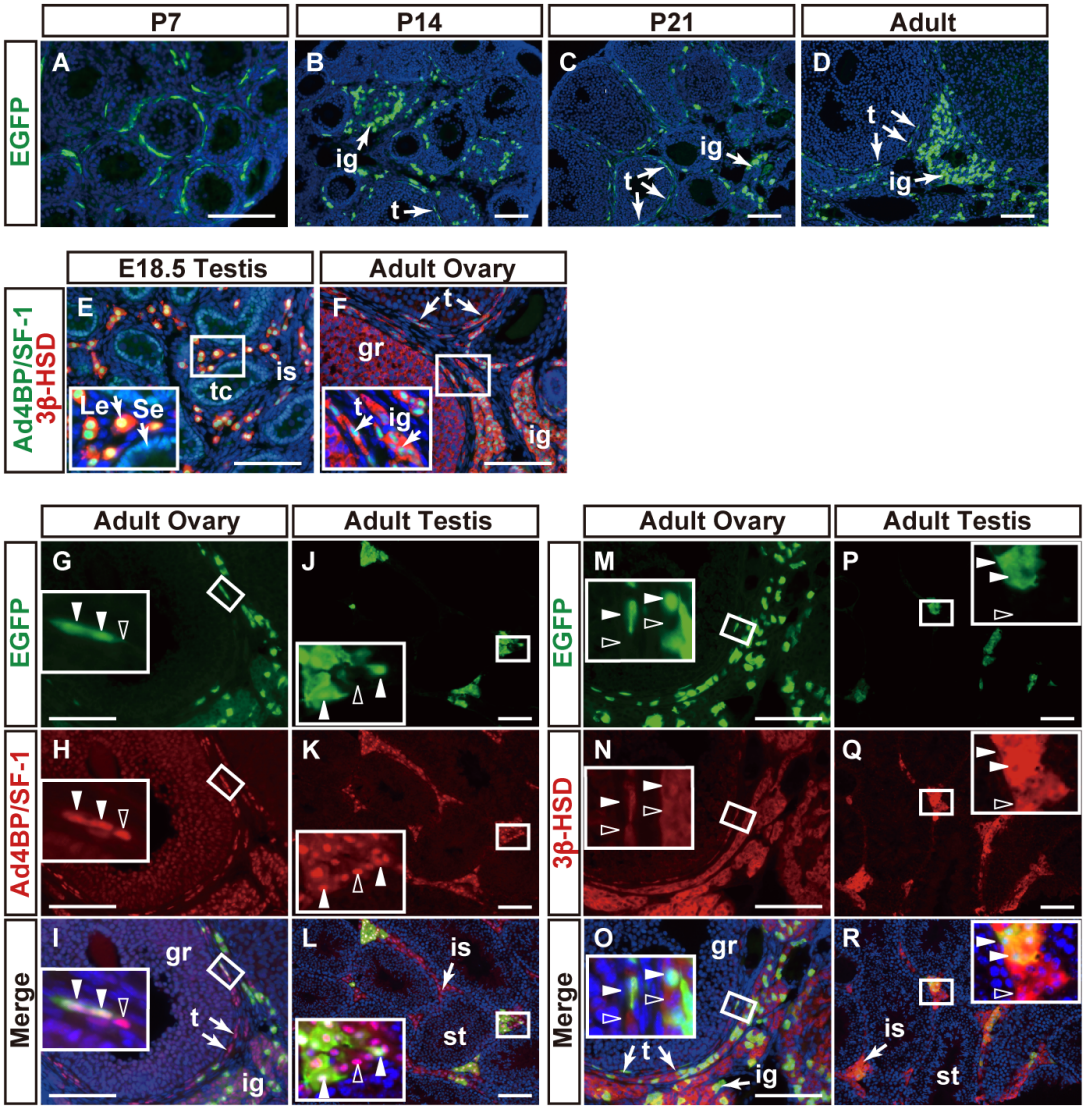
Distribution of EGFP-positive cells in mFLE-EGFP gonads. mFLE-EGFP transgenic mouse ovaries at P7 (A, n = 4), P14 (B, n = 2), P21 (C, n = 4), and adult stage at P42 or P56 (D, F, G-I, M-O, n = 4), and testes at E18.5 (E) and adult stage at P42 or P56 (J-L, P-R) were sectioned and subjected to immunofluorescence with antibodies for EGFP (green), Ad4BP/SF-1 (green in E, and red in others), and 3β-HSD (red). Merged images for Ad4BP/SF-1 and 3β-HSD (E, F), EGFP and Ad4BP/SF-1 (I, L), and EGFP and 3β-HSD (O, R) are shown. A-F, I, L, O, and R were further stained with DAPI (blue). Arrows in E indicate Sertoli cells (Se) or Leydig cells (Le). Arrows in F, I, L, O, and R indicate theca cells (t) or the interstitial gland (ig). Closed white arrowheads in G-L indicate cells double positive for EGFP and Ad4BP/SF-1, while those in M-R indicate cells double positive for EGFP and 3β-HSD. Open arrowheads in G-L indicate single positive cells for Ad4BP/SF-1, while those in M-R indicate single positive cells for 3β-HSD. Insets are enlarged views of the areas enclosed by rectangles. t, theca cells; ig, interstitial gland; gr, granulosa cells; Le, Leydig cell; Se, Sertoli cell; tc, testicular cord; is, interstitial space. Scale bars = 100 μm.

The expression of EGFP in the developing ovaries was examined using immunofluorescence. EGFP-positive cells were localized sporadically surrounding early-stage follicles at P7 (Fig 3A). The same pattern was seen surrounding the developing follicles at P14 and P21. In addition, there were EGFP-positive cells in the stromal region (Fig 3B and 3C). In the cycling ovary at the adult stage, the distribution of EGFP-positive cells surrounding follicles was essentially similar to that at the former stage, and EGFP-positive cells were assembled in the stromal region (Fig 3D). The stromal region of mammalian ovaries such as rodents has been discovered to contain steroidogenic cells, and thus the region has been referred to as an interstitial gland. In summary, our observations strongly suggested that FLE is activated in not all, but rather a proportion of steroidogenic theca and interstitial gland cells in the ovary. 16.4±1.6% of Ad4BP/SF-1-positive cells were positive for EGFP in the adult ovary.

Ad4BP/SF-1 and 3β-HSD (3β-hydroxysteroid dehydrogenase) were used as markers for steroidogenic cells in the gonads. Indeed, Ad4BP/SF-1 and 3β-HSD were detected immunohistochemically in the interstitial cells of the fetal testes, and the signals were observed to overlap completely. The expression of Ad4BP/SF-1 but not 3β-HSD was detected weakly in Sertoli cells (Fig 3E). Similar to in the testis, the strong signals of Ad4BP/SF-1 and 3β-HSD overlapped in the theca and interstitial gland cells in the adult ovary (Fig 3F). Additionally, the expression of 3β-HSD was detected weakly in the granulosa cells, while that of Ad4BP/SF-1 was undetectable in the follicles. Regulation of *Ad4BP/SF-1* gene expression in developing follicular granulosa cells is still controversial. Its expression appears to be detectable in some follicles but undetectable in others. This difference is unlikely due to developmental stage of the follicle because similar size between follicles does not necessarily mean similar levels of expression of Ad4BP/SF-1, even though they may appear healthy but not atretic. Considering that *Hsd3b1* gene expression is regulated by Ad4BP/SF-1, the absence of Ad4BP/SF-1 from the follicle where 3β-HSD is expressed suggests that a transcription factor other than Ad4BP/SF-1 would be involved in *Hsd3b1* gene regulation. In this regard, it is interesting to note that LRH-1 (NR5A2), which potentially binds to the same sequence as Ad4BP/SF-1 does, regulates *Cyp19* gene expression in ovarian granulosa cells [30, 31]. Likewise, LRH-1 in place of Ad4BP/SF-1 may regulate *Hsd3b1* in such follicles, as shown in Fig 3F.

The expression of EGFP in the adult ovaries of mFLE-EGFP transgenic mice was compared with those of endogenous Ad4BP/SF-1 and 3β-HSD. As shown in Fig 3G–3I, the staining for EGFP overlapped with those for Ad4BP/SF-1 in the ovarian theca and interstitial gland cells. However, interestingly, Ad4BP/SF-1-positive cells were not always positive for EGFP, and EGFP single positive cells were not observed. When the expression of 3β-HSD was examined, a partially overlapped distribution between EGFP and 3β-HSD was observed again (Fig 3M–3O). These results suggested that theca and interstitial gland cells consist of at least two different cell types: FLE is activated within one of them but not activated within the other.

As previously described, the expression of EGFP could be observed even in postnatal testes of mFLE-EGFP mice [19]. This persistent expression of EGFP was compared with those of Ad4BP/SF-1 and 3β-HSD in adult testes. As expected, both Ad4BP/SF-1 and 3β-HSD are expressed in all interstitial Leydig cells, while EGFP was expressed in a population of Ad4BP/SF-1 (Fig 3J–3L) and 3β-HSD positive cells (Fig 3P–3R), indicating that FLCs are present in the adult testis. In conclusion, the gonads of the two sexes seems similar from the aspect that the steroidogenic cells consist of two cell types in terms of their enhancer usage of *Ad4BP/SF-1* gene.

### Comparison of reporter gene expressions driven by FLE and the whole genomic region of the *Ad4BP/SF-1* gene

**Fig 4:**
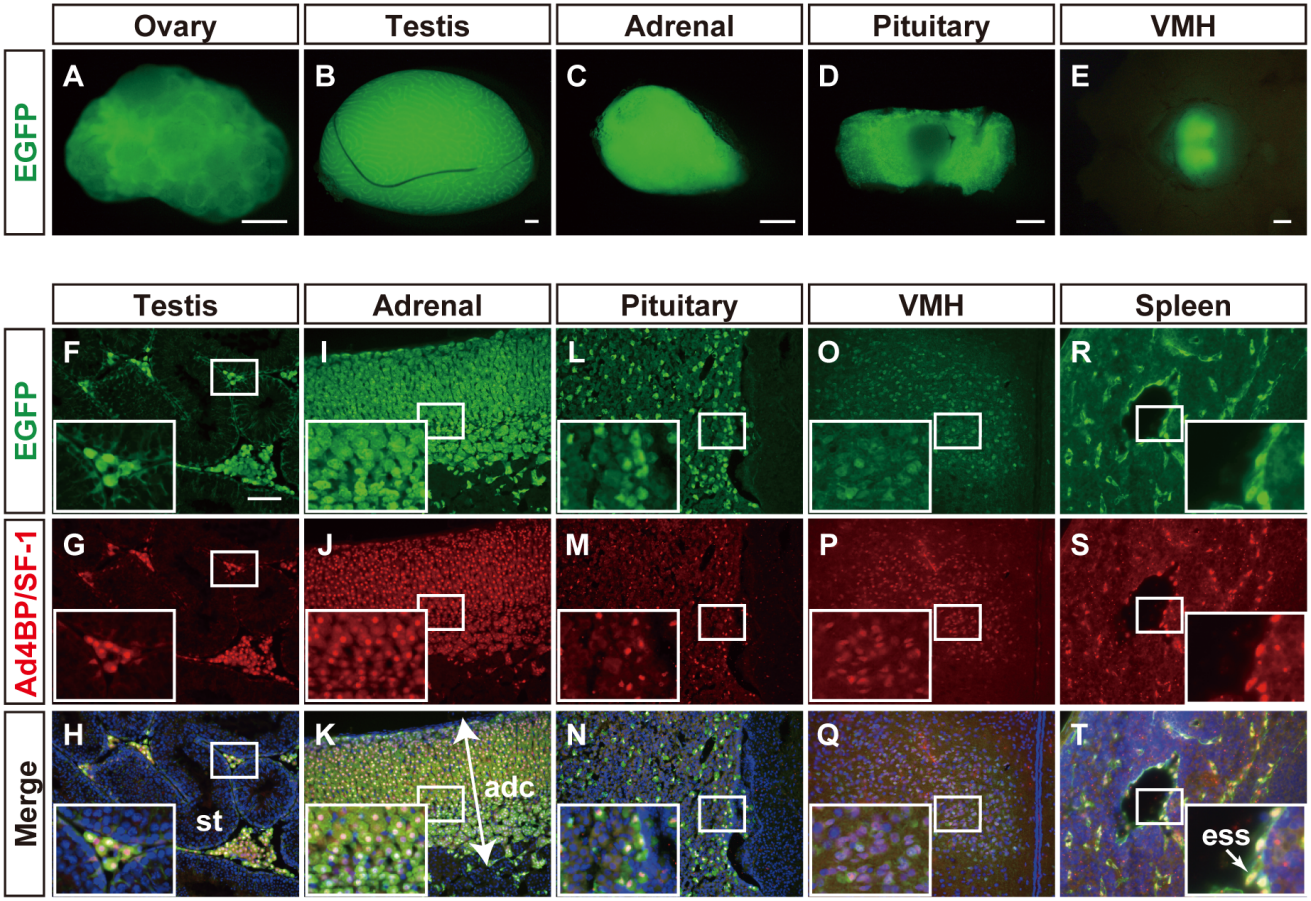
Expression of EGFP induced by Ad4BP-BAC-EGFP. The ovary (A), testis (B), adrenal gland (C), pituitary (D), and VMH (E), in which endogenous *Ad4BP/SF-1* is expressed, were prepared from adult Ad4BP-BAC-EGFP transgenic mice (n = 3). EGFP expression was observed under a fluorescent microscope. The testis (F-H), adrenal gland (I-K), pituitary (L-N), VMH (O-Q), and spleen (R-T) were sectioned, followed by immunofluorescence with antibodies for EGFP (green in F, I, L, O, and R) and Ad4BP/SF-1 (red in G, J, M, P, and S). Merged views of EGFP and Ad4BP/SF-1 are shown in H, K, N, Q, and T, which are further stained with DAPI (blue). Insets are enlarged views of the areas enclosed by rectangles. st, seminiferous tubule; adc, adrenal cortex; ess, endothelial cell of splenic sinus. Scale bars in A-E = 500 μm and those in F-T = 100 μm.

Subsequently, we attempted to compare the reporter gene expression driven by FLE with that by the BAC clone containing the *Ad4BP/SF-1* gene locus. Reporter gene expression driven by the BAC construct (Ad4BP-BAC-EGFP described in Materials and Methods) was expected to recapitulate endogenous *Ad4BP/SF-1* gene expression, and indeed, as expected, EGFP expression was observed in the ovary, testis, adrenal gland, pituitary, and ventromedial hypothalamus (VMH) by fluorescence microscopy (Fig 4A–4E). These results are consistent with those previously obtained using a yeast artificial chromosome as an expression vector [32]. Immunohistochemical experiments for Ad4BP/SF-1 demonstrated the EGFP expression overlapped with endogenous Ad4BP/SF-1 in the testis (Fig 4F–4H), adrenal cortex (Fig 4I–4K), pituitary (Fig 4L–4N), and VMH (Fig 4O–4Q). Although a clear signal was not detected by fluorescent microscopic in the spleen, expression in the endothelial cells of the splenic sinus was identified by immunofluorescence [33] (Fig 4R–4T).

**Fig 5:**
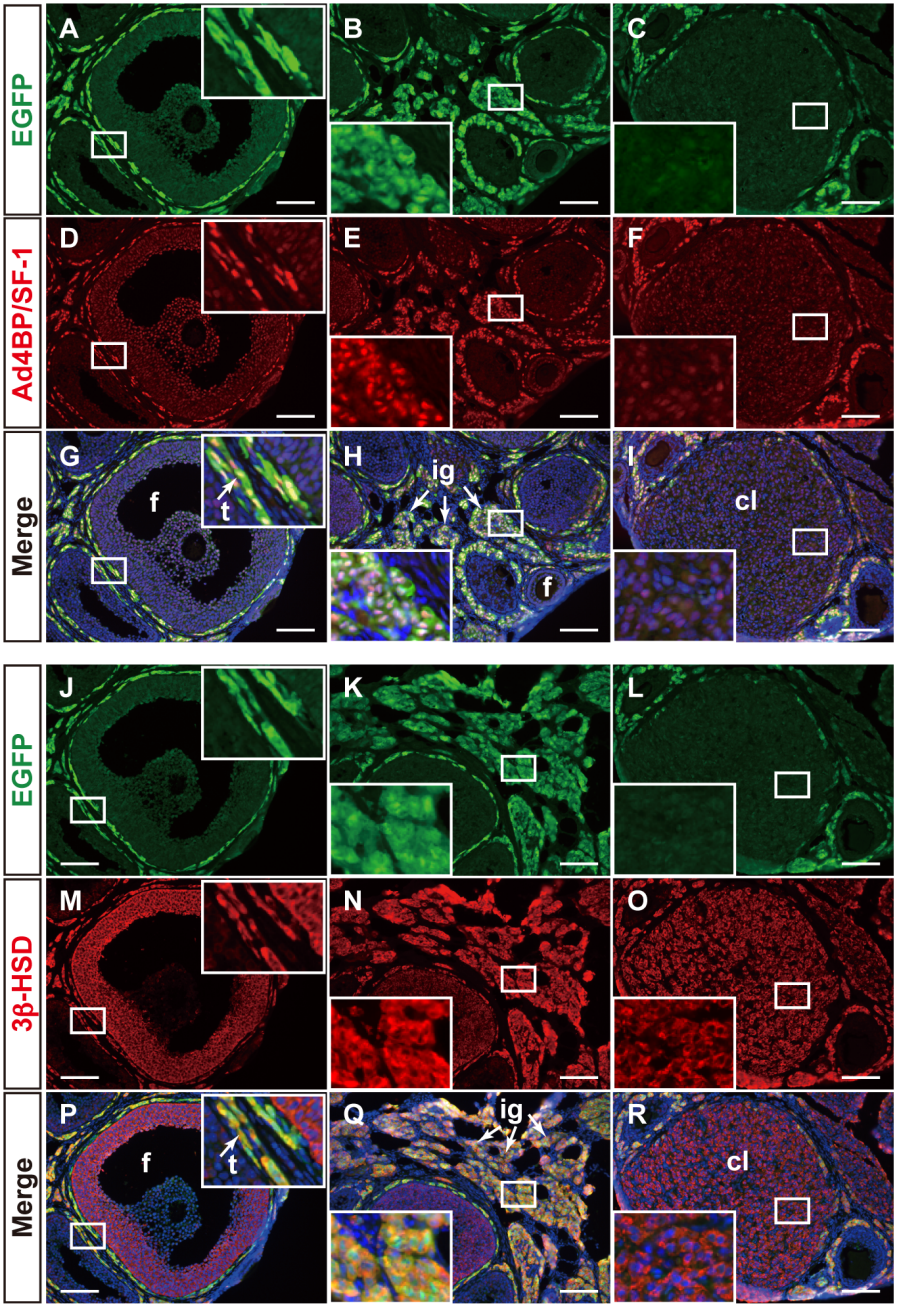
Distribution of EGFP-positive cells in Ad4BP-BAC-EGFP ovary. The adult ovaries at P42 or P56 were prepared (n = 3) and subjected to immunofluorescence with the antibodies for EGFP (green in A-C and J-L), Ad4BP/SF-1 (red in D-F), and 3β-HSD (red in M-O). Merged views for EGFP and Ad4BP/SF-1 are shown in G, H and I, while those for EGFP and 3β-HSD are shown in P, Q, and R; these are stained simultaneously with DAPI (blue). Arrows in G, H, P and Q indicate theca cells (t) or the interstitial gland (ig). Insets are enlarged views of the areas enclosed by rectangles. f, follicle; t, theca cells; ig, interstitial gland; cl, corpus luteum. Scale bars = 100 μm.

The ovarian expression of EGFP in the BAC transgenic mice was investigated immunohistochemically. Strong EGFP expression was observed in the theca cells surrounding well-developed antral follicles (Fig 5A), preantral follicles (Fig 5B), and corpora lutea (Fig 5C). Likewise, interstitial gland cells were strongly stained with EGFP (Fig 5B). In addition to these relatively strong expression signals, weaker expression was observed in granulosa cells and luteal cells (Fig 5A–5C). The distribution and strength of these expressions were consistent with the endogenous expression of Ad4BP/SF-1 (Fig 5D–5I). The expression of 3β-HSD was detected in theca cells, interstitial gland cells, and granulosa cells (with the exception of cumulus cells) of well-developed follicles, and luteal cells (Fig 5M–5O). The expressions of 3β-HSD in theca and interstitial gland cells overlapped well with those of EGFP, while the expressions of 3β-HSD in granulosa and luteal cells were stronger than those of EGFP (Fig 5J–5L and 5P–5R).

**Fig 6:**
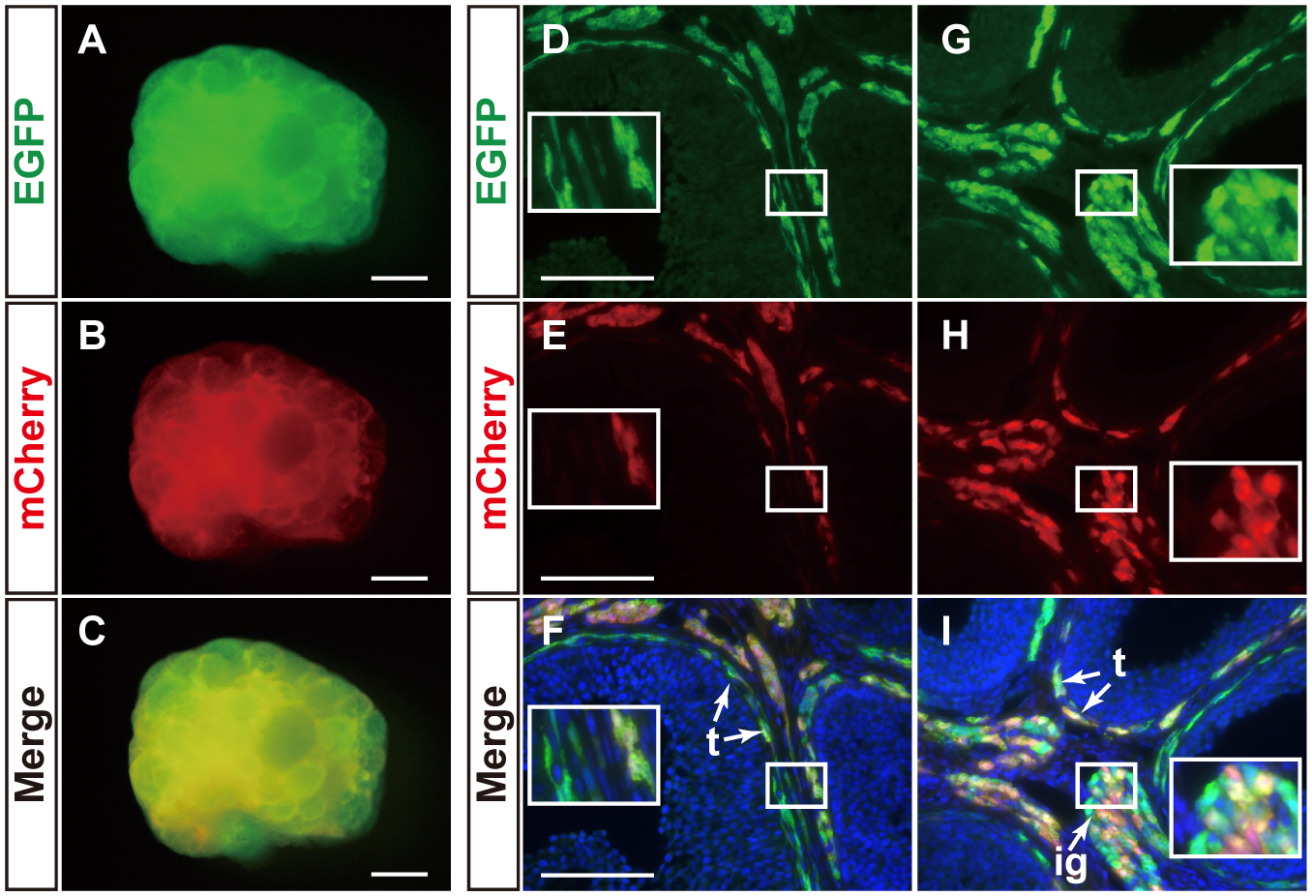
Overlapped expression of EGFP driven by BAC-Ad4BP and mCherry by mFLE. Ad4BP-BAC-EGFP transgenic mice were crossed with mFLE-mCherry transgenic mice to generate double transgenic mice (n = 3). Fluorescence views of the adult ovary are shown (A-C). The ovaries were subjected to immunofluorescence with the antibodies for EGFP (green in D and G) and mCherry (red in E and H). Merged views of EGFP and mCherry are shown in F and I, which are further stained with DAPI (blue). Arrows in F and I indicate theca cells (t) or the interstitial gland (ig). Insets are enlarged views of the areas enclosed by rectangles. t, theca cells; ig, interstitial gland. Scale bars for A-C = 500 μm and those for D-I = 100 μm.

To compare the cells labeled with the BAC and mFLE constructs, we additionally established transgenic mouse line using *mCherry* as the reporter gene (mFLE-mCherry). Ad4BP-BAC-EGFP and mFLE-mCherry mice were crossed, and the resulting ovaries carrying the two transgenes were subsequently examined. The ovaries displayed fluorescence for EGFP and mCherry (Fig 6A–6C). To localize precisely the mutual distribution of the transgenes, the adult ovaries were subjected to immunohistochemical analyses for EGFP and mCherry. As shown in Fig 6D–6I, all mCherry-positive theca and interstitial gland cells were positive for EGFP recapitulating endogenous Ad4BP/SF-1 expression, while the EGFP-positive cells were not always positive for mCherry. mCherry single positive cells were not observed.

## Discussion

It has been established that mammals develop fetal and adult types of steroidogenic cells in the adrenal gland (the fetal and adult adrenocortical cells) [22, 34, 35] and testis (fetal and adult Leydig cells) [14, 19, 36]. In rodents, the fetal adrenal cortex starts to disappear around birth, and by puberty onset has completely disappeared in males, whereas it disappears completely during the first pregnancy in females [37]. Likewise, it has been suggested that fetal Leydig cells (FLCs) are replaced by adult Leydig cells (ALCs) in the early postnatal period, as discussed below. However, the ovaries are different from the male gonad, in that steroidogenic cells are not yet differentiated during the fetal period; indeed, no fetal theca and interstitial gland cells with an appearance resembling FLCs have thus far been reported.

We previously identified FLE in the *Ad4BP/SF-1* gene, and thereby established transgenic mice in which FLCs are labeled with EGFP (mFLE-EGFP) [20]. Tracing the labeled cells unexpectedly revealed that EGFP-positive FLCs are present until the adult period. This persistent presence of FLCs in the adult testis of rodents has been suggested by morphological studies to date [38, 39]. Different from the testis, the ovary was believed to not harbor any fetal type of steroidogenic cells. Nevertheless, the present study demonstrated that EGFP-positive cells, in which FLE is activated, emerge in the postnatal ovaries. Immunohistochemical experiments suggested that these cells are a population of theca and interstitial gland cells. This finding was supported by comparing the expression of a reporter gene driven by an *Ad4BP/SF-1* BAC clone (Ad4BP-BAC-EGFP). Based on the observations, it is highly likely that theca and interstitial gland cells consist of at least two cell populations, in one of which the FLE of *Ad4BP/SF-1* is activated, while in the other a yet-unknown enhancer is. This differential enhancer usage between the two cell types bears strong resemblance to that between FLCs and ALCs, although the enhancer for ALCs has yet to be characterized.

Although it has not been reported that the ovarian theca and interstitial gland cells consist of multiple cell types in mammals, the presence of two types of theca cells has been described in the ovaries of medaka fish: one type expresses *cyp17* while the other expresses *cyp19* [40]. Considering the differential expression of the steroidogenic genes, the former cells seem to synthesize either androstenedione or testosterone as the final product, and the latter seem to synthesize estradiol. However, there has not been any report on mammals showing the *Cyp19* gene to be expressed in theca and interstitial gland cells. Therefore, it is unlikely that the two types of theca cells found in this study correspond to those found in medaka fish. Nevertheless, it is still interesting to note that two types of theca cells have been found in these two evolutionarily distant animals.

Because we currently do not have any marker gene to distinguish these two cell types, it remains unclear whether other mammalian species develop them. Additionally, it is still unclear whether they are functionally differentiated or not. Addressing these issues would further our understanding of the functional significance of the two theca and interstitial gland cell types in the ovary.
